# Auditory Brainstem abnormalities in infants exposed to congenital TORCH infections: implications for longitudinal audiological monitoring

**DOI:** 10.1007/s00431-026-07543-9

**Published:** 2026-10-22

**Authors:** Glauciene Amaral Martins, Liliane Aparecida Fagundes Silva, Carla Gentile Matas

**Affiliations:** Department of Physical Therapy, Speech-Language Pathology and Occupational Therapy, School of Medicine, University of São Paulo, São Paulo, Brazil

**Keywords:** Congenital Infections, Auditory Evoked Potentials, Auditory Pathways, Infant, Longitudinal Studies

## Abstract

To evaluate the integrity and maturation of the auditory pathway in infants exposed to congenital infections by comparing them with infants without risk indicators for hearing loss. This prospective longitudinal study included 55 infants exposed to congenital TORCH infections (study group—SG) and 38 infants without risk indicators for hearing loss (control group—CG) enrolled in a Neonatal Hearing Screening program at a public hospital in São Paulo, Brazil. Infants exposed to congenital TORCH infections and a control group without risk indicators for hearing loss were evaluated. Participants underwent three assessments: before 3 months of age (T1), 3 months after the first assessment (T2), and 6 months after the first assessment (T3). Auditory Brainstem Response (ABR) and Long-Latency Auditory Evoked Potentials (LLAEP) were recorded. Absolute latencies and interpeak intervals of the ABR, as well as the P1 component latency of the LLAEP, were analyzed. Infants exposed to congenital infections showed a higher frequency of ABR abnormalities involving waves III and V, interpeak intervals I–III and I–V, and the overall ABR outcome, particularly at the initial assessments. Progressive reductions in ABR wave and interpeak latencies, as well as in LLAEP P1 latency, were observed over time in both groups. No significant between-group differences were found in P1 latency.

*Conclusion:* Infants exposed to congenital infections exhibit alterations in brainstem auditory pathway maturation, suggesting a slower or distinct maturational trajectory than infants without risk indicators for hearing loss. Longitudinal audiological follow-up is essential for the early identification of auditory pathway abnormalities in this population.

**Table:** 

**What is known:**
• *Congenital infections may impair auditory development by affecting the peripheral and central auditory pathways.*
**What is new:**
• *Infants exposed to congenital TORCH infections showed a higher frequency of Auditory Brainstem Responses abnormalities, suggesting a slower or distinct brainstem auditory maturation trajectory.*

## Introduction

Infections acquired during pregnancy may adversely affect both maternal and fetal health, leading to significant neonatal sequelae. One of the major complications associated with congenital infections is sensorineural hearing loss (SNHL), which may result from inflammatory damage to cochlear cells or direct cytotoxic effects of the infectious agent [1]. In addition to cochlear involvement, retrocochlear abnormalities have also been reported [2].

The congenital infections most commonly associated with hearing impairment are collectively referred to by the acronym TORCH, which includes toxoplasmosis, other infections (syphilis, hepatitis B, Zika virus, and human immunodeficiency virus—HIV), rubella, cytomegalovirus (CMV), and herpes simplex virus [3].

Although the pathophysiology of SNHL associated with congenital infections is not yet fully understood, evidence suggests that infectious agents may directly damage cochlear hair cells or trigger inflammatory and immune responses, cellular necrosis, and vascular alterations within the inner ear. These mechanisms may affect the cochlea, auditory nerve, and brainstem, ultimately disrupting central nervous system (CNS) development. During embryogenesis, viral infections such as CMV and HIV may also interfere with membranous labyrinth formation, cochlear differentiation, and the expression of critical genes involved in auditory development, including *GATA3, EYA1, SIX1,* and *POU4F3*. Furthermore, even in the absence of direct viral invasion of the cochlea, maternal and fetal immune activation may contribute to auditory damage through inflammatory pathways [1, 4].

The maturation of the auditory pathway can be objectively assessed using Auditory Brainstem Responses (ABR), which evaluate the integrity of the peripheral auditory system and brainstem auditory pathways, and Long-Latency Auditory Evoked Potentials (LLAEP), whose P1 component is a well-established biomarker of thalamocortical auditory maturation during the first years of life [5–7].

Given the importance of auditory maturation for early childhood development, infants with risk indicators for hearing loss (RIHL) should undergo not only conventional Neonatal Hearing Screening (NHS) but also electrophysiological assessments such as ABR to evaluate the central auditory pathway [3]. Furthermore, because congenital infections are associated with progressive or late-onset SNHL, longitudinal audiological monitoring is recommended.

Electrophysiological assessment provides valuable information on auditory pathway development in infants exposed to congenital infections during intrauterine life and may enable the early detection of CNS abnormalities affecting auditory function. These findings may contribute to improved audiological monitoring strategies and enhance the understanding of the effects of congenital TORCH infections on auditory development.

We hypothesized that infants exposed to congenital infections would be more likely to exhibit delayed maturation of the auditory pathway than infants without risk indicators for hearing loss. Therefore, the aim of this study was to evaluate the integrity and longitudinal maturation of the auditory pathway in infants exposed to congenital TORCH infections.

## Materials and methods

### Study design

This prospective longitudinal study was approved by the Institutional Research Ethics Committee of the Hospital das Clínicas, Faculdade de Medicina, Universidade de São Paulo (HCFMUSP) (approval No. 6.724.329).

### Participants

The study population consisted of infants enrolled in an NHS program at a public hospital in São Paulo, Brazil.

The study group (SG) comprised 55 infants younger than 3 months at the first assessment who had been exposed to congenital TORCH infections, with exposure confirmed by maternal and neonatal serological testing, who passed the NHS, and whose parents or legal guardians provided written informed consent. Infants with a family history of hearing loss, neurological disorders, syndromes, maternal illicit drug use during pregnancy, or type B or C tympanograms at any assessment were excluded.

The control group (CG) included 38 infants younger than 3 months at the first assessment, without RIHL, who passed the NHS and whose parents or legal guardians provided written informed consent. Infants with type B or C tympanograms at any assessment were excluded.

### Study procedures

Medical records were initially reviewed to identify infants who met the eligibility criteria. Parents or legal guardians of eligible participants were contacted and informed about the study objectives and procedures.

Participants were evaluated at three time points: before 3 months of age (T1), 3 months after the first assessment (T2), and 6 months after the first assessment (T3).

At T1, written informed consent was obtained, followed by medical history collection to confirm eligibility criteria and obtain relevant clinical information.

Otoscopy was performed using a Mini Heine 2000 otoscope (Heine, Herrsching, Germany) to identify any conditions that could interfere with audiological testing.

At all three assessments, audiological evaluation began with immittance measurements using a Titan system (Interacoustics, Middelfart, Denmark). Tympanometry was performed using a 1-kHz probe tone, followed by ipsilateral acoustic reflex testing at 1 kHz to assess middle ear integrity. Infants presenting abnormal middle ear findings were referred for otorhinolaryngological evaluation.

Transient Evoked Otoacoustic Emissions (TEOAE) were subsequently recorded using the same equipment with a pass/fail protocol and nonlinear click stimuli presented at 80 dB SPL. A passing result required a signal-to-noise ratio greater than 6 dB in at least three of the five frequency bands analyzed [8]. This procedure was performed to confirm normal cochlear function.

ABR recordings were obtained in an electrically and acoustically treated room using a two-channel Navigator Pro system (Biologic, Natus Medical Inc., USA) with ER-3A insert earphones while the infant was naturally asleep. After skin preparation with abrasive paste, electrodes were placed according to the International Electrode System [9], with the active electrode positioned at Fz and the reference electrodes at M1 and M2. Electrode impedance was maintained below 7 kΩ, with an interelectrode difference not exceeding 3 kΩ [10]. Rarefaction click stimuli were presented monaurally at 80 dB nHL at a rate of 13.3 stimuli/s using a 100–3000 Hz band-pass filter. A total of 2,000 stimuli were delivered within a 24-ms analysis window. Two replicated waveforms were obtained for each ear to ensure waveform reproducibility. Absolute latencies of waves I, III, and V, as well as interpeak intervals I–III, III–V, and I–V, were analyzed. Latencies were compared with age-specific normative values according to corrected age at the time of assessment and classified as normal or abnormal [11]. The overall ABR result was considered abnormal when prolonged absolute latency of any wave and/or interpeak interval was identified in either ear.

LLAEP recordings were subsequently obtained using the same equipment and electrode montage while infants remained naturally asleep. Tone-burst stimuli (1 kHz) were presented monaurally at 70 dB nHL at a rate of 1.1 stimuli/s, with a 5-ms rise time and 10-ms fall time, using a 1–100 Hz band-pass filter. A total of 200 stimuli were presented within a 512-ms analysis window. The latency of the P1 component, defined as the first prominent positive peak occurring at approximately 100 ms, was analyzed.

### Statistical analysis

Statistical analyses were performed using JAMOVI version 2.6.44. Both within-group and between-group analyses were conducted. Continuous variables are presented as mean ± standard deviation, whereas categorical variables are presented as frequencies and percentages.

Categorical outcomes were compared using the chi-square test, with Yates’ continuity correction applied when appropriate. Effect sizes were estimated using the Phi coefficient (φ) or Cramér's V and interpreted as small (0.1), medium (0.3), or large (0.5). Age and gestational age was compared between groups using the Mann–Whitney U test.

Statistical significance was set at *p* < 0.05.

## Results

A total of 93 infants were enrolled at baseline (SG = 55; CG = 38). Participant retention was 37/55 (67.3%) in the SG and 32/38 (84.2%) in the CG at the second assessment, and 32/55 (58.2%) and 29/38 (76.3%), respectively, at the third assessment. The clinical and demographic characteristics of the study population at baseline are presented in Table 1.

**Table 1:** Sample characterization according to clinical and demographic variables in the study and control groups

Variable	SG (*N* = 55)	CG (*N* = 38)	*p*-value
**Quantitative variables**	**mean ± SD (min–max)**	**mean ± SD (min–max)**	
Gestational age (weeks)	38.60 ± 1.10 (36ー40)	38.32 ± 1.40 (36ー40)	.390
Age at first assessment (weeks)	4.02 ± 2.22 (1ー9)	5.03 ± 2.70 (1ー10)	.079
Age at second assessment (weeks)	17.81 ± 3.07 (13ー25)	20.09 ± 4.23 (13ー32)	.014*
Age at third assessment (weeks)	32.72 ± 3.90 (28ー41)	34.76 ± 4.53 (27ー47)	.059
**Qualitative variables**	**N (%)**	**N (%)**	
Female sex	26 (47.27%)	23 (60.53%)	.295
Male sex	29 (52.73%)	15 (39.47%)	
AGA	42 (76.36%)	37 (97.37%)	.023*
SGA	4 (7.27%)	0 (0.00%)	
disprop SGA	8 (14.55%)	0 (0.00%)	
LGA	1 (1.82%)	1 (2.63%)	
Toxoplasmosis exposure	16 (29.10%)	0 (0.00%)	—
Syphilis exposure	19 (34.50%)	0 (0.00%)	
HIV exposure	6 (10.90%)	0 (0.00%)	
CMV exposure	14 (25.50%)	0 (0.00%)	

Quantitative variables are presented as mean ± standard deviation (minimum–maximum) and were compared using the Mann–Whitney U test. Qualitative variables are presented as n (%) and were compared using the chi-square (χ^2^) test. *p < 0.05 indicates statistical significance. *SG* study group, *CG* control group, *SD* standard deviation, *min* minimum, *max* maximum, *AGA* appropriate for gestational age, *SGA* small for gestational age, *dispro SGA* disproportionate small for gestational age, *LGA* large for gestational age

Among infants in the SG, maternal infection occurred during the first trimester of pregnancy in 38 cases (69.1%), during the second trimester in three (5.5%), and during the third trimester in nine (16.4%). In five cases (9.1%), the timing of maternal infection could not be determined.

The proportions of normal and abnormal ABR findings according to age-specific normative values are presented in Table 2. As no significant differences were observed between the right and left ears, data from both ears were pooled for analysis.

**Table 2:** Analysis of ABR findings according to age-specific normative values in the study and control groups

Variable	Study group		Control group			Statistical test		
	Normal	Abnormal	Normal	Abnormal		χ^2^(1)	*p*	ϕ
First assessment								
Wave I	55 (100%)	0 (0.00%)	38 (100%)	0 (0.00%)	⊘		⊘	⊘
Wave III	45 (81.82%)	10 (18.18%)	38 (100%)	0 (0.00%)	5.963		.015*	.289
Wave V	45 (81.82%)	10 (18.18%)	38 (100%)	0 (0.00%)	5.963		.015*	.289
Interpeak I-III	41 (74.55%)	14 (25.45%)	38 (100%)	0 (0.00%)	9.483		.002*	.350
Interpeak III-V	53 (96.36%)	2 (3.64%)	38 (100%)	0 (0.00%)	0.213		.645	.123
Interpeak I-V	45 (81.82%)	10 (18.18%)	38 (100%)	0 (0.00%)	5.963		.015*	.289
Overall result	39 (70.91%)	16 (29.09%)	38 (100%)	0 (0.00%)	11.387		<.001*	.379
Second assessment								
Wave I	37 (100%)	0 (0.00%)	32 (100%)	0 (0.00%)	⊘		⊘	⊘
Wave III	29 (78.38%)	8 (21.62%)	32 (100%)	0 (0.00%)	5.859		.015*	.337
Wave V	32 (86.49%)	5 (13.51%)	32 (100%)	0 (0.00%)	2.868		.090	.260
Interpeak I-III	27 (72.97%)	10 (27.03%)	32 (100%)	0 (0.00%)	8.051		.005*	.383
Interpeak III-V	36 (97.30%)	1 (2.70%)	32 (100%)	0 (0.00%)	< 0.001		>.999	.113
Interpeak I-V	31 (83.78%)	6 (16.22%)	32 (100%)	0 (0.00%)	3.824		.051	.287
Overall result	26 (70.27%)	11 (29.73%)	32 (100%)	0 (0.00%)	9.208		.002*	.405
Third assessment								
Wave I	32 (100%)	0 (0.00%)	29 (100%)	0 (0.00%)	⊘		⊘	⊘
Wave III	27 (84.38%)	5 (15.63%)	29 (100%)	0 (0.00%)	3.078		.079	0.284
Wave V	28 (87.50%)	4 (12.50%)	29 (100%)	0 (0.00%)	2.108		.147	.252
Interpeak I-III	27 (84.38%)	5 (15.63%)	29 (100%)	0 (0.00%)	3.078		.079	.284
Interpeak III-V	31 (96.88%)	1 (3.13%)	29 (100%)	0 (0.00%)	< 0.001		>.999	.123
Interpeak I-V	28 (87.50%)	4 (12.50%)	29 (100%)	0 (0.00%)	2.108		.147	.252
Overall result	26 (81.25%)	6 (18.75%)	29 (100%)	0 (0.00%)	4.102		.043*	0.314

⊘—Chi-square test value was not estimated because all Wave I results were within normal limits; *p-value <.05

**Fig. 1:**
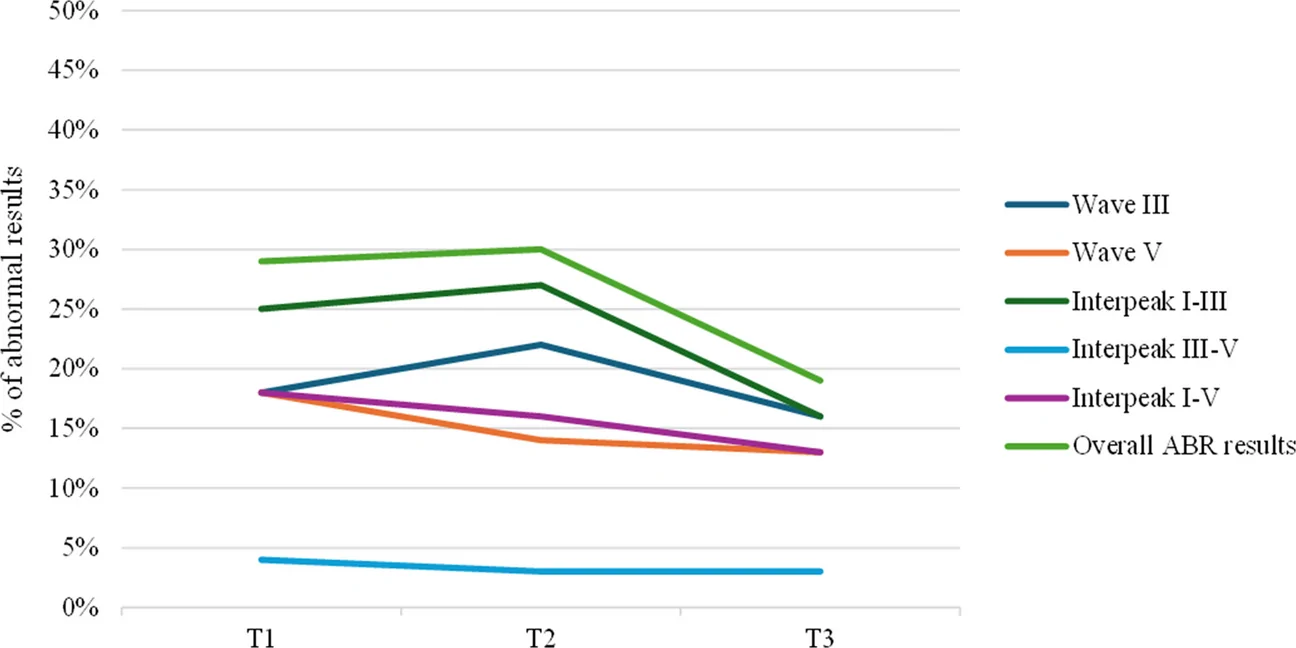
Proportion of abnormal ABR results for study group across the three assessments. T1 = first assessment; T2 = second assessment; T3 = third assessment; % = percentage

At T1, the SG showed a higher frequency of abnormalities than the CG for wave III, wave V, interpeak intervals I–III and I–V, and the overall ABR outcome. At T2, the SG continued to show a higher frequency of abnormalities for wave III, interpeak interval I–III, and the overall ABR outcome. At T3, a higher proportion of abnormal findings was observed only for the overall ABR outcome in the SG (Fig. 1).

Absolute latencies of waves I, III, and V, interpeak intervals I–III, III–V, and I–V, as well as P1 latency, progressively decreased over time in both groups. Table 3 summarizes the descriptive analysis of ABR and LLAEP latencies at each assessment. As no significant differences were found between the right and left ears, mean values from both ears were used for data presentation.

**Table 3:** Descriptive analysis of ABR latencies and interpeak intervals, and LLAEP latencies for each group across the three assessments

Variable	Study group mean ± SD (min–max)	Control group mean ± SD (min–max)
**First assessment**	*N* = 55	*N* = 38
Age (weeks)	4.02 ± 2.22 (1ー9)	5.03 ± 2.70 (1ー10)
Wave I	1.66 ± 0.100 (1.45ー1.95)	1.65 ± 0.096 (1.37ー1.87)
Wave III	4.42 ± 0.243 (3.95ー5.12)	4.32 ± 0.161 (3.87ー4.62)
Wave V	6.60 ± 0.283 (6.03ー7.62)	6.49 ± 0.162 (6.20ー6.87)
Interpeak I-III	2.76 ± 0.227 (2.25ー3.42)	2.67 ± 0.140 (2.33ー2.92)
Interpeak III-V	2.18 ± 0.152 (1.92ー2.50)	2.17 ± 0.115 (1.92ー2.42)
Interpeak I-V	4.94 ± 0.268 (4.50ー5.92)	4.84 ± 0.146 (4.50ー5.17)
LLAEP P1	227.48 ± 59.36 (109.00ー423.93)	206.50 ± 54.35 (106.42ー343.78)
**Second assessment**	*N* = 37	*N* = 32
Age (weeks)	17.81 ± 3.07 (13ー25)	20.09 ± 4.23 (13ー32)
Wave I	1.62 ± 0.081 (1.45ー1.78)	1.59 ± 0.073 (1.37ー1.70)
Wave III	4.21 ± 0.239 (3.75ー4.87)	4.10 ± 0.150 (3.78ー4.37)
Wave V	6.28 ± 0.257 (5.65ー6.87)	6.18 ± 0.152 (5.85ー6.45)
Interpeak I-III	2.60 ± 0.225 (2.20ー3.08)	2.51 ± 0.130 (2.25ー2.75)
Interpeak III-V	2.06 ± 0.156 (1.83ー2.58)	2.08 ± 0.111 (1.83ー2.33)
Interpeak I-V	4.66 ± 0.239 (4.10ー5.20)	4.59 ± 0.141 (4.22ー4.83)
LLAEP P1	175.03 ± 31.25 (107.00ー232.00)	155.87 ± 39.03 (77.28ー250.08)
**Third assessment**	*N* = 32	*N* = 29
Age (weeks)	32.72 ± 3.90 (28ー41)	34.76 ± 4.53 (27ー47)
Wave I	1.59 ± 0.086 (1.45ー1.78)	1.57 ± 0.073 (1.37ー1.62)
Wave III	4.07 ± 0.196 (3.62ー4.53)	3.97 ± 0.154 (3.70ー4.28)
Wave V	6.07 ± 0.180 (5.53ー6.45)	5.99 ± 0.155 (5.70ー6.28)
Interpeak I-III	2.48 ± 0.194 (2.08ー3.00)	2.41 ± 0.130 (2.17ー2.67)
Interpeak III-V	1.99 ± 0.112 (1.75ー2.33)	2.02 ± 0.097 (1.83ー2.17)
Interpeak I-V	4.48 ± 0.173 (4.08ー4.83)	4.42 ± 0.137 (4.17ー4.67)
LLAEP P1	131.86 ± 32.96 (67.91ー225.00)	112.70 ± 32.06 (68.95ー188.66)

*SD* standard deviation, *min* minimum, *max* maximum

## Discussion

The present study longitudinally investigated the integrity of the auditory pathway in infants exposed to congenital infections by comparing them with age-specific normative parameters and with infants without RIHL.

Initially, the groups showed a similar distribution regarding gestational age, sex, and age at the first and third assessments, suggesting homogeneity between the SG and CG for most clinical and demographic variables. However, a difference was observed in age at the second assessment, with the CG being evaluated at a later time point. This difference should be considered when interpreting the results.

Only infants classified as appropriate for gestational age were included in the CG, whereas a higher proportion of small-for-gestational-age infants was observed in the SG. Being born small for gestational age is an important indicator of impaired fetal growth, defined as birth weight below the 10th percentile for gestational age, and is associated with adverse perinatal and long-term outcomes [12]. Therefore, the higher frequency of small-for-gestational-age infants in the SG was expected, as congenital infections may contribute to fetal growth restriction, prematurity, congenital malformations, and developmental abnormalities [13].

In the SG, exposure to syphilis was the most frequent, followed by toxoplasmosis, CMV, and HIV, reflecting the epidemiological profile of the population followed at our institution [14].

The results showed a progressive reduction in the absolute latencies of ABR waves and interpeak intervals, as well as in the P1 component latency of the LLAEP, in both groups. These findings suggest continuous maturation of the peripheral and central auditory pathways throughout development, allowing greater neural efficiency through physiological processes such as myelination, increased neural synchrony, and refinement of synaptic connections, which are particularly pronounced during the first months of life [15, 16].

However, infants exposed to congenital infections showed a higher frequency of ABR abnormalities, particularly during the first two assessments, whereas no abnormalities were observed in the CG. The differences were more evident for waves III and V, interpeak intervals I–III and I–V, and the overall ABR outcome. Wave I remained within age-appropriate normative values throughout all assessments. Although the frequency of abnormalities decreased over time, the SG continued to show a higher frequency of abnormal findings up to the third assessment.

The absence of ABR abnormalities in the CG across all assessments may be partly related to the strict eligibility criteria adopted for this group, which included the absence of risk indicators for hearing loss. This stringent selection resulted in a highly selected low-risk population and may have contributed to the restricted variability observed in the CG. Therefore, the absence of abnormalities in the CG should be interpreted within the context of this selected population and should not be considered representative of the full range of auditory outcomes that may occur among infants without identified risk factors.

These findings indicate that auditory conduction remained within the expected range up to the auditory nerve, the generator site of wave I. However, slower neural conduction was observed between the distal portion of the auditory nerve and the cochlear nucleus or superior olivary complex, the generator sites of wave III, as reflected by the prolonged I–III interpeak interval. In addition, the upper brainstem also showed evidence of delayed conduction, since wave V reflects neural activity within the lateral lemniscus. Finally, the I–V interpeak interval represents the conduction time from the auditory nerve to the lateral lemniscus, and its prolongation suggests generalized slowing of neural conduction throughout the brainstem auditory pathway [7].

This pattern suggests longer neural conduction times at central levels of the auditory pathway. Furthermore, the progressive reduction in the frequency of these abnormalities throughout follow-up suggests continuity of the maturational process, reinforcing the existence of ongoing neural reorganization and plasticity during the first years of life, although following a slower or distinct trajectory compared with infants not exposed to congenital infections [15, 16]. Therefore, these findings appear to reflect a delay or difference in the maturational trajectory of the auditory pathway rather than permanent impairment of the involved structures.

The presence of these abnormalities may be related to the inflammatory, infectious, or neurotropic effects associated with congenital infections, which may interfere with CNS development during critical periods of gestation. Alterations in myelination, synaptic organization, or brainstem maturation may contribute to distinct developmental trajectories, even in the absence of evident peripheral hearing impairment [1, 17].

The reduction in P1 latency has been described as an objective marker of auditory cortex maturation, reflecting increased neural processing efficiency, myelination, and refinement of cortical connections throughout development [15, 18, 19]. The absence of differences between groups in the P1 component contrasts with the ABR findings and may suggest that the impact of congenital infections on auditory maturation in the present sample was more evident at subcortical levels or during the early stages of neural conduction, whereas cortical auditory maturation remained similar between groups. It is important to emphasize that, because the study was conducted in infants, the analysis of the LLAEP was limited to the P1 component, since the remaining components emerge later during development [18, 19].

These findings are consistent with those of Hora et al. [20], who also reported no abnormalities in the P1 component in children prenatally exposed to Zika virus. However, those authors observed an increased P2 amplitude, which is associated with auditory discrimination and attention and may indicate cortical auditory immaturity. Since the P2 component emerges only between 6 and 8 years of age [18, 19], it could not be evaluated in the present study.

The findings of the present study reinforce the importance of longitudinal audiological follow-up in children exposed to congenital infections, even in the absence of evident peripheral hearing abnormalities or after initial results within the expected range [3]. The persistence of ABR abnormalities in some infants suggests that single assessments, including NHS alone, may not be sufficient to identify late-onset abnormalities or differences in central auditory pathway development. Therefore, children exposed to congenital infections may benefit from continuous audiological monitoring, allowing the early identification of auditory abnormalities and timely intervention during periods of greater neural plasticity [21].

Future studies investigating the impact of these findings on the development of auditory and language skills are warranted. Previous studies have shown that prolonged wave III and V latencies and reduced wave V amplitude on ABR may be observed in children with learning and language disorders [22], and that the maturational process assessed by ABR may predict subsequent language development even in typically developing children [23]. Therefore, the early identification of delayed auditory pathway maturation may be crucial for developing early intervention strategies and improving audiological and educational follow-up.

The present study has some limitations. The heterogeneity of congenital infections included in the SG, the small number of participants in some etiological subgroups, and losses to follow-up throughout the study may have limited the statistical power of the longitudinal analyses and the comparison among specific etiologies. In addition, a difference in age between the groups was observed at the second assessment. Despite these limitations, the longitudinal design allowed the evaluation of the maturational trajectory of the auditory pathway over time, contributing to the understanding of the possible effects of congenital infections on auditory development during infancy.

In conclusion, infants exposed to congenital infections showed a higher frequency of ABR abnormalities, particularly involving waves III and V and interpeak intervals I–III and I–V, with these differences being most evident at the initial assessments, when compared with age-specific normative values and the CG. Although a progressive reduction in latencies and in the frequency of abnormalities was observed throughout follow-up, the findings suggest a slower or distinct maturational trajectory of the brainstem auditory pathway in these infants. In contrast, the P1 component of the LLAEP showed a similar developmental pattern in both groups, indicating preserved early cortical maturation. These findings reinforce the importance of longitudinal audiological follow-up in children exposed to congenital infections for the early identification of abnormalities in auditory pathway development.

## Data Availability

The datasets generated and/or analyzed during the current study are available from the corresponding author on reasonable request.
